# Effects of temperature, hyposalinity, and diminishing sperm concentration on fertilisation and embryonic development in *Acropora tumida* and *Platygyra carnosa*

**DOI:** 10.1038/s41598-026-41257-0

**Published:** 2026-03-20

**Authors:** Taison Ka Tai Chang, Jenny Tsz Ching Chan, Billy Chun Ting Cheung, Gerard F. Ricardo, Peter J. Mumby, Apple Pui Yi Chui

**Affiliations:** 1 Simon F.S. Li Marine Science Laboratory, School of Life Sciences, The Chinese University of Hong Kong, Hong Kong SAR, China; 2 Marine Spatial Ecology Lab, School of the Environment, The University of Queensland, St Lucia, Brisbane, QLD 4072 Australia; 3 Institute of Environment, Energy and Sustainability, The Chinese University of Hong Kong, Hong Kong SAR, China; 4 State Key Laboratory of Marine Pollution, City University of Hong Kong, Kowloon Tong, Hong Kong SAR, China

**Keywords:** Climate change, Coral early life stages, Marginal coral populations, Thermal stress, Salinity, Sperm availability, Marine biology, Climate-change ecology

## Abstract

Evaluating climate change impacts, for example, temperature anomaly and hyposalinity, on coral reproduction across a diversity of systems, including high-latitude marginal environments, is important. Here, we examined temperature and hyposalinity impacts on fertilisation under various sperm concentrations, and embryo development of two locally common hard corals, *Acropora tumida* and *Platygyra carnosa*, in Hong Kong. A salinity of 22 psu reduced fertilisation success by ~ 83% for both species. Our results demonstrated that increasing sperm concentration will be required to achieve the same amount of fertilisation success (i.e. 50%) under decreasing salinity. Temperature had species-specific effects on fertilisation. Decreased fertilisation was recorded under both lowered (– 3 °C) and elevated temperature (+ 3 °C) for *A. tumida*. For *P. carnosa*, fertilisation decreased under lowered temperature but increased under elevated temperature. For embryonic development, 26 psu salinity resulted in 33 and 40% more abnormal embryos for *A. tumida* and *P. carnosa*. The effects of temperature on embryonic development again varied between species. Under ambient salinity, elevated temperature led to 60% more abnormal embryos for *P. carnosa*, but only 8% for *A. tumida*. With reef degradation, diminishing sperm abundance will interact with rising climate change stressors and potentially impair coral reproduction.

**Supplementary Information:**

The online version contains supplementary material available at 10.1038/s41598-026-41257-0.

## Introduction

Broadcast spawning corals reproduce by shedding eggs and sperm into the water column, where external fertilisation occurs. Successful fertilisation and subsequent survival and development of embryos are critical life-history stages. Any adverse impact on these stages may result in a bottleneck during recruitment^1^. Maximum fertilisation success typically requires sperm concentrations of 10^5^–10^6^ sperm mL^–1^^2^. However, following spawning, the concentrations of gametes in the water column decline rapidly due to natural dilution^2^. In addition, changes in the environment and water chemistry can further reduce effective sperm concentrations, thereby limiting the success of external fertilisation and embryonic development^3–5^. To overcome these obstacles, marine broadcast spawners often have developed strategies to enhance fertilisation success, such as synchronous spawning^6^, or spawning in periods of minimum rainfall^7,8^ and low wind^9^. Yet, global warming will likely bring unprecedented extreme climate events, for example, marine heatwaves, more intense rainstorms and typhoons, that may alter these patterns and result in the catastrophic failure of coral reproduction.

Coral fertilisation and early embryonic development are highly sensitive to elevated sea surface temperatures (SST), with responses varying among species^10–13^. For example, in Okinawa, a 4 °C increase in SST resulted in an 80% reduction in fertilisation success for *Acropora millepora*, whereas no negative effects were observed in *Favites chinensis* under the same conditions^11^. Elevated temperatures can also increase the chance of having abnormally developed coral embryos^11–13^. For example, at Davies Reef on the Great Barrier Reef, a 4 °C increase in SST led to a 40% increase in abnormal development of *A. millepora* embryos, whereas no effects were observed for *Favites abdita* and *Mycedium elephantotus*^11^. Nevertheless, thermal stress is not the lone stressor under predicted climate scenarios. The intensity of cyclones and rainstorms is predicted to increase with the changing climate^14,15^. This increases the chances of having extreme climate events, such as rainstorms or cyclones, that overlap with coral spawning events. Under these circumstances, coral fertilisation and embryogenesis can occur under an abrupt reduction in salinity^3,10,12,16,17^. Fertilisation was found to be entirely suppressed, corresponding to a 100% reduction, for *Acropora millepora* under the salinity of 28 psu^17^. Similarly, decreased salinity often leads to higher rates of abnormal embryonic development in multiple coral species^3,10,12,16^. A study in Hong Kong has investigated the combined effects of elevated temperature and hyposalinity, and revealed an antagonistic effect, with elevated temperature extending the range of tolerance to reduced salinity in *Platygyra acuta*^10^. Specifically, elevated temperature enhanced normal early embryonic development at a lowered salinity of 26 psu, and this antagonistic interaction was consistently observed across two successive nights of spawning^10^. Recent transcriptomic analyses on *Acropora pruinosa* have further shown that the molecular pathways enriched under elevated temperature and hyposalinity are largely distinct^18^, highlighting the complex and potentially interactive nature of these stressors on coral reproductive success.

Coral reefs are increasingly degraded under climate change and coral communities are increasingly fragmented^19,20^. The reduction in adult colony density decreases sperm availability for broadcast spawning corals and increases susceptibility to Allee effects, which compromises fertilisation success^21,22^. Additionally, the already limited sperm availability can further decrease when spawning overlaps with major rainstorms. Experiments using sperm concentrations of ~ 10^5^–10^6^ sperm mL^–1^(see^2^ and^23^), may be unrealistically high for many reefs subject to hydrodynamic dispersive forces or in coral communities with lower coral population sizes, for example those growing under marginal coral environments^24–26^. Thus, results from studies using a single sperm concentration and long egg-sperm contact times (e.g. >3 h) when examining the effects of temperature or salinity on coral fertilisation may not reflect the true field scenarios^3,10,12,27^. Experimental designs that incorporate multiple sperm concentrations can more reliably estimate the size of a stressor’s effect, provide better insights into mechanisms involved, and increase comparability between studies^28^. Such designs have been used to assess the effects of ocean acidification, temperature, sediments, and nutrients, on coral fertilisation^29–31^.

**Fig. 1:**
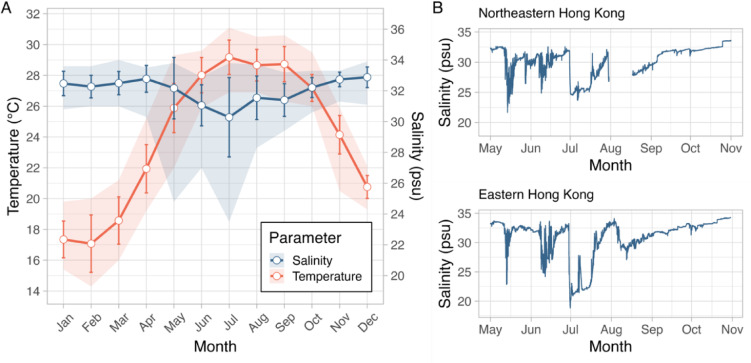
Historical sea surface temperature and salinity records of Hong Kong in the last two decades. (**A**) Monthly mean sea surface salinity records from 2002 to 2021 in one of the water monitoring stations (MM5) in northeastern Hong Kong, which is the closest station to our coral sampling site (Tung Ping Chau Marine Park). Data were collected by the Environmental Protection Department of the HKSAR Government. The red and blue solid lines show the mean sea surface temperature and salinity, respectively, over the 20-year period. The shaded areas show the maximum and minimum temperature or salinity recorded in the 20-year period. (**B**) Daily sea surface salinity from May to Oct 2022, recorded by the Agricultural, Fisheries and Conservation Department of the HKSAR Government, in two monitoring stations in northeastern (Wong Wan) and eastern (Kau Sai) Hong Kong.

Hong Kong, located in northern South China Sea, is characterised by having large seasonal fluctuations in sea surface temperatures and salinity^32,33^(Fig. 1A). In the South China Sea region, more marine heatwaves have been documented in the recent decade^34^, and the intensity and duration of such events are projected to increase through the 21st century^35^. With global warming, elevated seawater temperature is predicted to have an increased chance to overlap with coral spawning, which can reduce early reproductive success and subsequent population recovery^36^. In Hong Kong, corals generally spawn in late spring to early summer, which often overlaps with the monsoonal rainstorm events^37^. The intensity of these extreme weather events in Hong Kong is expected to be more severe as projected from downscale climate modelling^38^. The seawater conditions could be highly variable, with fluctuation in temperature and salinity that could impact early coral reproductive processes such as fertilisation and embryonic development^10,12,18^. For example, in Hong Kong, a 3 °C increase in SST had no negative effect on fertilisation success in *Platygyra acuta*, but resulted in an approximate 40% decline in normal embryonic development^10^. In addition, in this species, fertilisation success was reduced by 60% at 24 psu, while the proportion of normally developed embryos decreased by more than 80% at 26 psu^10^. Specifically, hyposalinity condition persisted for over 24 h during coral spawning monitoring in 2014 in Hong Kong, which could overlap entirely with gamete fertilisation and early embryonic development^10^. And during the early summer of 2022, following a severe typhoon, salinity anomalies of 22 and 25 psu (compared to an average of ~ 30 psu) were recorded for approximately two weeks at Wong Wan (northern Hong Kong) and Kau Sai (northeastern Hong Kong), where corals are abundant (~ 22−75% coral cover) (Fig. 1B). Hong Kong, therefore, provides a realistic testing ground for examining the combined effects of temperature and salinity stresses on the early reproductive stages of corals from a marginal environment.

Here, we assessed the combined effects of temperature and salinity stress on (1) fertilisation success and (2) embryogenesis of two common coral species *Acropora tumida* and *Platygyra carnosa*, from a marginal coral habitat, Hong Kong. Using a range of sperm concentrations in our experimental design, we aim to (3) determine the effective sperm concentration necessary to ensure field fertilisation success of the two coral species. To our knowledge, our study is the first to determine the combined effects of temperature and salinity on coral fertilisation across a range of realistic sperm concentrations. Our findings can provide better insights into the possible consequences of diminishing sperm concentration under future climate change scenarios on coral recruitment.

## Results

**Fig. 2:**
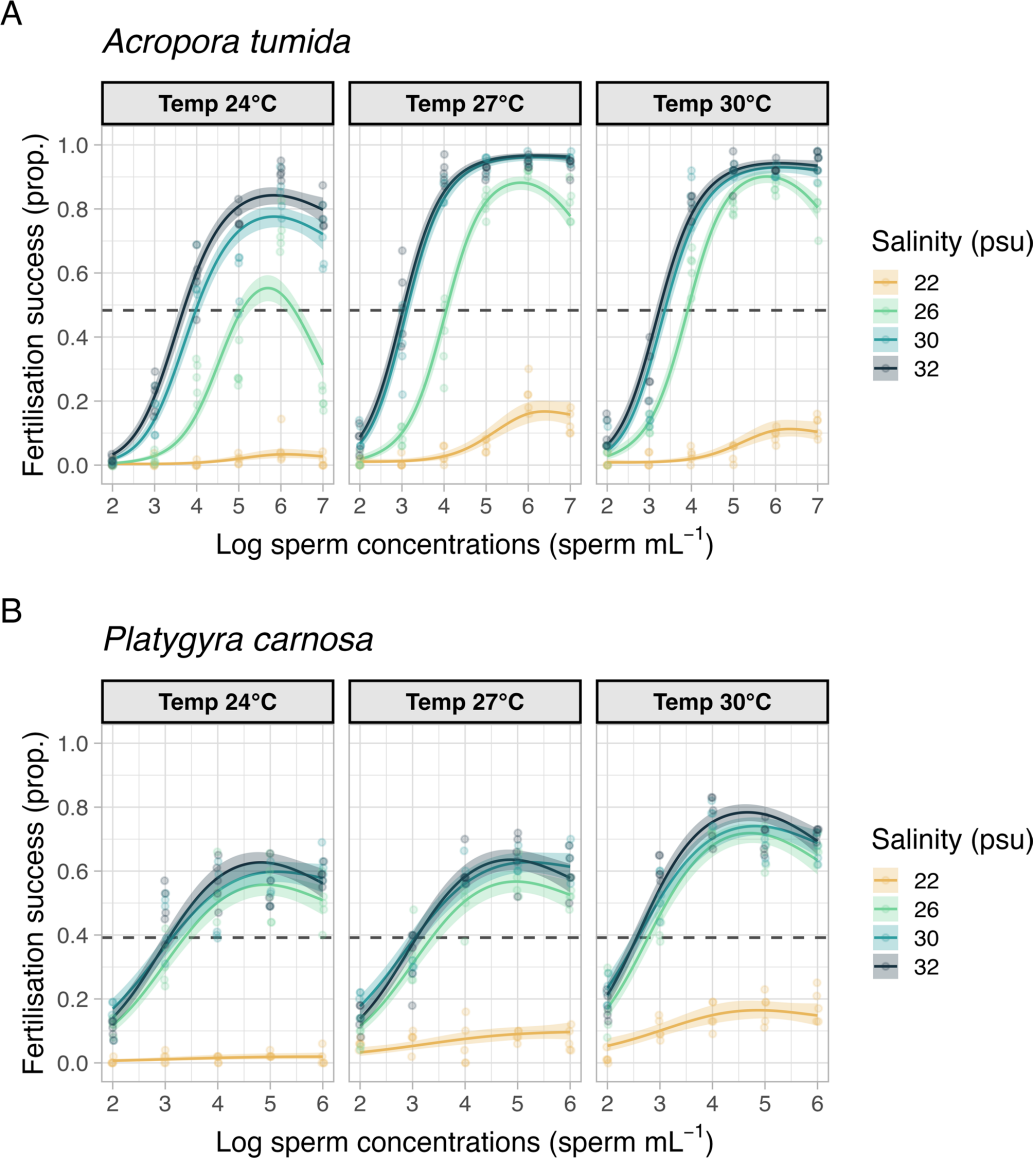
Fertilisation success (proportion) of (**A**) *Acropora tumida* and; (**B**) *Platygyra carnosa* under three temperatures, 24, 27 (ambient temperature), 30 °C and four salinity treatment levels 22, 26, 30, 32 (ambient salinity) psu, across a range of sperm concentrations (10^2^ to ≥ 10^6^ sperm mL^− 1^). Colour lines represent generalised additive mixed model fits. Shaded areas represent 95% confidence intervals. Colour dots represent raw data points. The dotted lines show 50% of the maximum fertilisation success among all the experimental treatments.

Effects of sperm concentrations on coral fertilisation success Decreasing sperm concentrations across all temperature and salinity treatments generally resulted in reduced fertilisation success. Notably, there was a decrease in fertilisation success under the highest sperm concentration (10^6^ and 10^7^ sperm mL^–1^ for *P. carnosa* and *A. tumida*, respectively) across all stress treatments (Fig. 2). The optimal sperm concentration for each species remained overall constant (10^5^ and 10^6^ sperm mL^–1^ for *P. carnosa* and *A. tumida*, respectively) across experimental treatments, whereas the maximum fertilisation declined.

### Effects of combined stressors on coral fertilisation success

Results from the statistical analysis using Generalised Additive Mixed Models (GAMM) revealed that the interaction of temperature and salinity, and the smoothing term of sperm concentration with temperature and salinity, were significant predictors for fertilisation success (*p* < 0.001 except the smoothing term of sperm concentration for *P. carnosa*, which was 0.005, Table S1). When we compared different salinity treatments in the post-hoc pairwise comparisons in both species (Table S2), low salinity of 22 psu resulted in significantly lower fertilisation success compared to those of the other three levels of salinity under all temperatures for both species (all comparison pairs with *p* < 0.001, Table S2). Under salinity of 26 psu, fertilisation success of *A. tumida* was significantly lower than that under salinity of 30 and 32 psu, regardless of temperature (*p* < 0.001, Table S2). Yet, the same salinity level of 26 psu did not result in significantly lower fertilisation success in *P. carnosa*. For salinity of 30 psu, only the fertilisation success of *A. tumida* under lowered temperature (24 °C) was significantly lower than that under ambient salinity (32 psu) (*p* < 0.001). When we compared the fertilisation success under different temperature treatments (Table S3), in both species, all temperature comparison pairs showed statistically significant differences (*p* < 0.001) in the fertilisation success except the pairs between 27 °C and 30 °C under the lowest (22 psu) and ambient salinity (32 psu).

The maximum fertilisation success (F_max_) for *A. tumida* reached a maximum of 96.3 ± 0.8% under ambient salinity and temperature (32 psu and 27 °C) (Fig. 2A; Table 1). For *P. carnosa*, F_max_ reached 78.0 ± 1.4%, under the elevated temperature of 30 °C and ambient salinity of 32 psu (Fig. 2B; Table 1) but only a peak of 63.4 ± 1.5% at ambient conditions of both temperature and salinity.

**Table 1:** Mean sperm concentrations required to achieve 50% of peak fertilisation (EC_50_) and maximum fertilisation success (F_max_) across different temperature and salinity treatments for the two coral species, *Acropora tumida* and *Platygyra carnosa*. The mean values and standard deviations (SD) of the EC_50_ and F_max_ at each treatment level were derived by resampling the residuals and refitting the GAMM 1000 times.

		*Acropora tumida*			*Platygyra carnosa*		
Temperature (°C)	Salinity (psu)	Mean EC_50_ ± SD (log sperm mL^–1^)		Mean F_max_ ± SD (%)	Mean EC_50_ ± SD (log sperm mL^–1^)		Mean F_max_ ± SD (%)
24	22	N.A.	5.45 ± 1.45		N.A.	4.58 ± 1.18	
24	26	5.09 ± 0.10	55.69 ± 2.25		3.36 ± 0.08	55.81 ± 1.56	
24	30	3.97 ± 0.07	77.45 ± 1.95		3.13 ± 0.09	60.22 ± 1.53	
24	32	3.66 ± 0.07	83.87 ± 1.80		3.07 ± 0.07	62.72 ± 1.57	
27	22	N.A.	16.75 ± 2.23		N.A.	10.66 ± 1.74	
27	26	4.06 ± 0.06	88.08 ± 1.31		3.34 ± 0.09	56.59 ± 1.57	
27	30	3.12 ± 0.05	95.81 ± 0.84		3.03 ± 0.08	62.81 ± 1.49	
27	32	3.03 ± 0.05	96.33 ± 0.77		3.05 ± 0.07	63.40 ± 1.53	
30	22	N.A.	12.05 ± 1.90		N.A.	17.09 ± 1.95	
30	26	3.92 ± 0.06	89.92 ± 1.27		2.76 ± 0.06	71.37 ± 1.49	
30	30	3.37 ± 0.06	92.78 ± 1.14		2.57 ± 0.07	73.86 ± 1.33	
30	32	3.23 ± 0.06	94.06 ± 1.05		2.54 ± 0.05	77.95 ± 1.44	

**Fig. 3:**
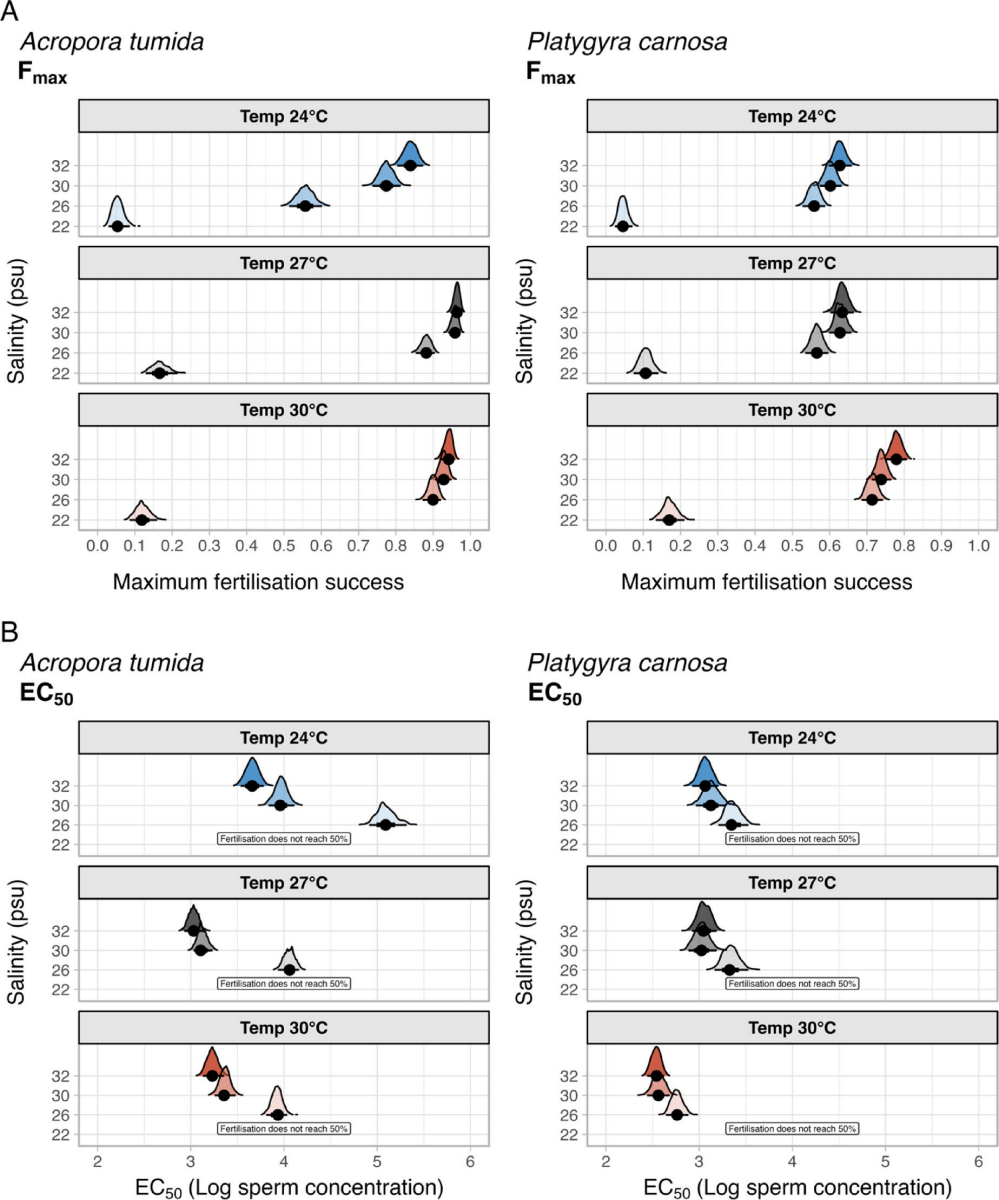
The bootstrapping results of the maximum fertilisation success (F_max_) and the sperm concentration required to achieve 50% of the maximum fertilisation (EC_50_). The F_max_ and EC_50_ parameters are extracted from the GAMM models on fertilisation success under three temperatures (24, 27, 30 °C) and four salinity treatment levels (22, 26, 30, 32 psu). The ridges represent the distributions of the bootstrapped values. Dots and whiskers below each distribution denote the mean and 66% (thick whiskers) and 95% (thin whiskers) confidence intervals.

Temperature significantly affects fertilisation success for both species (χ^2^ = 35.97, *p* < 0.001 for *A. tumida*; χ^2^ = 81.84, *p* < 0.001 for *P. carnosa*) (Table S1). For *A. tumida*, F_max_ was compromised under both low (24 °C) and elevated (30 °C) temperatures, where F_max_ decreased to 83.9 ± 1.8% and 94.1 ± 1.1% (Fig. 3A; Table 1), respectively, compared to 96.3 ± 0.8% at ambient temperature (27 °C) and salinity (32 psu). For *P. carnosa*, F_max_ was 63.4 ± 1.5% under ambient temperature and salinity, and was 62.7 ± 1.6% under low (24 °C) temperature and ambient salinity (Fig. 3B; Table 1). Fertilisation success of *P. carnosa* was enhanced and peaked at 78.0 ± 1.4% under elevated temperature of 30 °C and ambient salinity (Fig. 3B; Table 1). EC_50_ for both species shifted slightly under the lower and elevated temperature treatments. Under ambient salinity of 32 psu, EC_50_ increased from 10^3.03^ sperm mL^–1^ under ambient control temperature, to 10^3.23^ sperm mL^–1^ at elevated, and 10^3.66^ sperm mL^–1^ at lowered temperature for *A. tumida*. For *P. carnosa*, EC_50_ was the lowest at 10^2.54^ sperm mL^–1^ under elevated temperature, while EC_50_ was at 10^3.05^ and 10^3.07^ sperm mL^–1^ under control and lowered temperatures, respectively.

Maximum fertilisation success significantly decreased towards lower salinity treatments (χ^2^ = 632.87, *p* < 0.001 for *A. tumida*; χ^2^ = 266.99, *p* < 0.001 for *P. carnosa*) (Table S1). For *A. tumida*, F_max_ decreased from 95.8 ± 0.5% to 19.1 ± 2.2% when the salinity was reduced from 32 psu (ambient) to 22 psu under the ambient temperature of 27 °C (Fig. 3A; Table 1). Similarly, for *P. carnosa*, F_max_ decreased from 62.3 ± 1.4% at the ambient salinity to 12.0 ± 1.6% at the salinity of 22 psu, under the ambient temperature (Fig. 3B; Table 1). The same pattern was observed under other temperature treatments (Fig. 3B; Table 1). EC_50_ values all increased with the lowering of salinity, except under the salinity of 30 psu in *P. carnosa* where EC_50_ was lower when compared to that under ambient control salinity. Under the lowest salinity treatment of 22 psu, fertilisation success did not reach 50% of the maximum, even at optimal sperm concentrations (10^5^ or 10^6^ sperm mL^–1^) for both species in the experiment (Fig. 3).

### Embryo development under temperature and salinity treatments

**Fig. 4:**
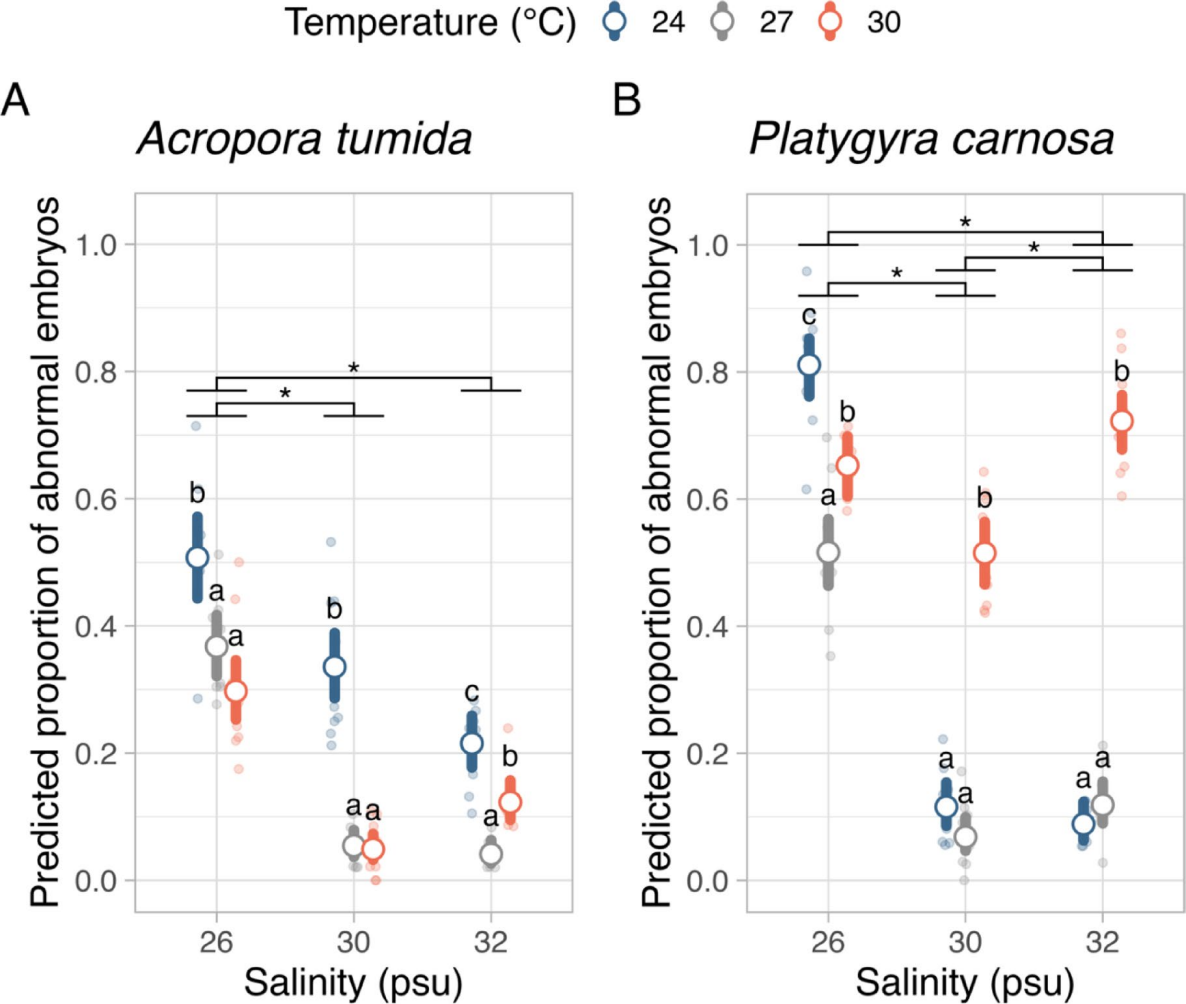
Proportions of abnormally developed embryos of (**A**) *Acropora tumida* and (**B**) *Platygyra carnosa*, under three temperature (24, 27, 30 °C) and three salinity (26, 30, 32 psu) treatments. The open points and lines represent the predicted mean and 95% confidence interval of the mean. Small, jittered dots show the raw data points. Small letters show statistically significant differences between temperature treatments within each salinity treatment level. Asterisks show statistically significant differences between salinity treatments when averaging the temperature levels.

The results of the effects of temperature and salinity on the proportions of abnormal embryos are shown in Fig. 4. Embryo development in both *A. tumida* (χ^2^ = 2.007, *p* = 0.157) and *P. carnosa* (χ^2^ = 0.662, *p* = 0.416) was not affected significantly by sperm concentrations (10^5^ vs. 10^6^ sperm mL^–1^. Therefore, data from the two sperm concentrations were pooled to examine the effect of temperature and salinity on embryo abnormality.

For both *A. tumida* and *P. carnosa*, temperature (χ^2^ = 153.45, *p* < 0.001 for *A. tumida*; χ^2^ = 354.75, *p* < 0.001 for *P. carnosa*), salinity (χ^2^ = 250.03, *p* < 0.001 for *A. tumida*; χ^2^ = 308.25, *p* < 0.001 for *P. carnosa*) and the interaction between the two stressors (χ^2^ = 63.64, *p* < 0.001 for *A. tumida*; χ^2^ = 268.60, *p* < 0.001 for *P. carnosa*) were significant factors in affecting the proportion of abnormal embryos that could be developed (Table S4). Post-hoc pairwise comparison results showed that low salinity treatment of 26 psu resulted in 32.7% significantly more abnormal embryos (*p* < 0.001) when compared to the control salinity of 32 psu, under 27 °C. For *A. tumida*, treatments with salinity of 30 and 32 psu at lowered temperature (24 °C) led to the development of 28.2 and 17.4% more abnormal embryos (*p* < 0.001 and < 0.05, respectively). In contrast, the same salinity treatments under elevated temperature of 30 °C did not increase the proportion of abnormal embryos developed. For *P. carnosa*, salinity of 26 psu again resulted in a significant 39.7% more abnormal embryos developed under the ambient temperature of 27 °C (*p* < 0.001). At salinities of 30 and 32 psu, elevated temperature (30 °C) resulted in the development of 44.7 and 60.4% more abnormal embryos (*p* < 0.001), while lowered temperature did not cause an increase in the proportion of abnormal embryos.

## Discussion

Climate change can lead to unpredictable sea surface temperatures and potential heavy rainstorms, which cause low salinity and diluted sperm during coral spawning. Our experiment mimicked the realistic stressful conditions for coral fertilisation under (1) cool or warm seawater temperatures, (2) reducing salinity and (3) diluting sperm concentration, co-occurring during coral spawning. We demonstrated that fertilisation success in *A. tumida* declined by 13% under the − 3 °C treatment, relative to the ambient temperature. Under a + 3 °C treatment relative to the ambient thermal condition, fertilisation of *A. tumida* was reduced, yet, that of *P. carnosa* was increased by 23%. Hyposalinity conditions of 26 psu significantly affected fertilisation, that we recorded a moderate decline of 9% in fertilisation success for *A. tumida*, and an 8% decline for *P. carnosa*. A salinity of 22 psu resulted in low fertilisation success of below 20% for both species, and that decreased to below 10% when coupled with lowered temperature of − 3 °C, even with optimal sperm concentration. In addition to impacts on fertilisation, we showed that temperature anomalies and reduced salinity could lead to higher proportions of abnormally developed coral embryos. While elevated temperature caused a 60% increase in abnormal embryonic development in *P. carnosa*, lowered temperature increased the proportion of abnormal embryos by 17% for *A. tumida*. Moreover, a low salinity of 26 psu caused 33% and 40% of abnormal embryos to develop in *A. tumida* and *P. carnosa*, respectively. Along with the low fertilisation success under hyposalinity conditions, the proportion of individuals that can continue to develop to the larval stage may become even more scarce, resulting in a bottleneck in coral recruitment^1^.

Thermal stress is one of the most studied stressors when estimating climate change impacts on coral fertilisation^11,12,39^, as the global oceans are predicted to continue to warm up in the coming decades, even with environmental remediation. A number of studies have focused on quantifying the potential loss in fertilisation success under elevated temperatures^11,39,40^. However, if coral spawning events overlap with heavy rainstorms, sea surface temperature is more likely to decrease rather than increase^41^. The lowered temperature treatment (– 3 °C) included in our study could therefore mimic this scenario more closely. Compared to warm conditions, we showed that a 3 °C reduction in temperature likely exerted more stress on the fertilisation of *A. tumida*. Based on studies of other marine invertebrates, fertilisation tended to have a narrow thermal breadth of optimal success, and decline towards lower and higher temperatures^42,43^. The decline of fertilisation under abnormal temperatures, both cool and warm conditions, can be due to the impairment of the enzymes involved in cell development and reduce fertilisation success^11^. While for *P. carnosa*, fertilisation success is higher under the + 3° treatment, suggesting that the thermal optimum for fertilisation in this species may be higher than the current ambient temperature of ~ 27 °C during coral spawning in Hong Kong. Indeed, a previous study in Hong Kong reported that reduced fertilisation success (76%) observed on the second day of spawning at ambient temperature in *Platygyra acuta* was recovered to 99% under an elevated temperature treatment (+ 3 °C)^10^, indicating a positive effect of increased temperature on fertilisation success. Notably, the average monthly sea temperature in Hong Kong has exhibited a slight upward trend over the past five years (Fig. S1), suggesting that the local condition is potentially shifting towards the higher optimum observed in *P. carnosa*. This difference in optimal temperatures for fertilisation success may drive varied recovery capacity for different coral species under future climate scenarios.

In addition to temperature anomalies, climate change-induced extreme rainfall events can create unfavourable conditions for coral fertilisation with a sharp reduction in sea surface salinity^3^, as well as a rapid dilution of gamete^2^. We show that a salinity of 22 psu alone, which is a realistic salinity level in the region during monsoon seasons, can potentially lead to an approximate 80% decline in fertilisation for both species. Hyposalinity stress can further be exacerbated when a large volume of rainwater accelerates sperm dilution, so that sperm concentration will likely fall outside the optimal range rapidly after spawning. The inclusion of multiple sperm concentrations can provide ecologically relevant perspectives and allow us to identify temperature and hyposalinity stressor effects by comparing the required sperm concentration to achieve the same level of fertilisation success. Our results demonstrated that increasing sperm concentration will be required to achieve the same amount of fertilisation success (i.e. 50%) under decreasing salinity. With future projection of reducing abundance of corals under climate change^19^, the shortage of sperm will potentially become a bottleneck for coral recovery for many degrading coral populations.

Decreasing salinity can potentially impair the motility of sperm, shown in studies on echinoderms^44^, oysters^45^, and fish^46,47^. On the other hand, hyposalinity may result in deterioration of eggs, and contribute to lowered fertilisation success, which has been reported in other marine invertebrates^48–50^. The inability to osmoregulate, therefore restricting water inflow and outflow into or from the cells, and the swelling of the hyaline layer due to osmotic stress may explain the deterioration of gametes^51^. From our results, interestingly, the optimal sperm concentration for each species remained overall constant across experimental treatments, whereas the maximum fertilisation declined. This downward shift of the fertilisation response curve is attributed to damage to the eggs rather than damage to the sperm or the sperm-egg interactions^28^. These findings contrast with those on most toxicants and stressors, which often report damage to the sperm as exhibited by the rightward shift of the fertilisation curve^29,31,52^.

Abnormality in coral embryogenesis is associated with various environmental stressors, including elevated temperature^13^, excessive nutrients^17^, and toxic heavy metals^53^. In our samples, the abnormal embryos were mainly severely deformed, including asymmetrical and irregular cleavages^11^, and had little chance to further develop into normal larvae. Therefore, we excluded the embryo abnormalities observed in our experiment in the case of polyembryony, which is considered to be a phenomenon that favours the reproduction of marine invertebrates by increasing the number of offspring through embryo fragmentation^54–56^. Two suggested pathways may cause irregular cell division. The first is deactivating the mitosis promoting factor (MPF) under reduced salinity. The lack of MPF leads to failure in cell division and embryonic cleavage^57^. Another possible mechanism of embryonic deformity is the loss of adhesion between cells. From a transcriptomic perspective, elevated temperature stress likely causes the downregulation of several genes that are responsible for regulating cell adhesion during coral embryonic stage^58^. The failure to establish cell adhesion will cause fragmentation in the developing embryos and result in asymmetric embryos^58^.

Marine broadcast spawners typically have lower sperm availability thresholds to fertilise eggs compared with other organisms^59^, but even between coral species, there are notable differences^23,60^. In general, both species examined in the present study have similar EC_50_ of 10^3^ sperm mL^–1^ under ambient temperature and salinity (Table 1). Considering the smaller egg size of *P. carnosa* compared to that of *A. tumida*, our results did not support the previous studies that showed higher fertilisation success under limited sperm concentration for organisms with larger eggs^61,62^. Theoretically, larger eggs would have an increased target surface area, thus a higher opportunity of encounter with the sperm to have a greater chance of being fertilised^62^. However, these studies often compared egg sizes and fertilisation success of conspecifics. The mechanism behind the larger the egg size, the higher the fertilisation success at lower sperm concentrations may not hold when we compare interspecific fertilisation. Another critical factor affecting fertilisation success is the proportion of motile sperm^63^. While the exact mechanism of how coral eggs of different coral species activate their conspecific sperm is not well understood, the proportion of motile sperm can vary between species and spawning nights^63,64^. In Hong Kong, coral communities are generally small (< a few hectares) with coral colonies of different genera relatively widely scattered. Although synchronised spawning of colonies of the same species does occur, the field sperm availability is likely to be naturally low. This low sperm availability threshold for achieving a relatively high fertilisation success in *Platygyra* species may potentially explain their dominance in Hong Kong^65^.

Notably, there was a decrease in fertilisation success when sperm concentrations reached 10^7^ and 10^6^ sperm mL^–1^ for *A. tumida* and *P. carnosa*, respectively, except under the ambient treatment for *A. tumida*. Similar observations have been reported in previous studies, primarily in non-acroporids^2,30,37^. For example, fertilisation of *Favites pentagona* and *Platygyra sinensis* reached their optima at the sperm concentration of about 10^5^ to 10^6^ sperm mL^–1^ and started to decline at higher concentrations^2^. Increased carbon dioxide produced by excessive sperm can contribute to lower dissolved oxygen and pH and result in lower fertilisation success^2^. Moreover, high sperm concentration may cause a breakdown of the polyspermy block^28,66^. This phenomenon has been described in several marine invertebrates^66–69^, when sperm concentrations are too high so that the eggs do not have enough time to activate polyspermy block after being contacted by one fertilising sperm, resulting in failure in fertilisation or embryogenesis^66^. Acroporids are considered to have a fast-block to polyspermy with rare declines in fertilisation success even at elevated sperm concentrations^23,70–72^, and here we observed similar trends in our ambient treatment controls. However, almost all the combinations of treatments resulted in an apparent decline in fertilisation at the higher sperm concentrations, indicating a possible breakdown of the efficient polyspermy block. Yet, the mechanism of polyspermy remains poorly understood in hard corals^70^. Future studies will be required to confirm the role and mechanism of polyspermy in coral fertilisation and how it interacts with other stressors.

Coral reproduction is the critical process for population persistence and recovery after disturbances. While most studies focused on the effect of climate change-related heat stress on coral reproduction^11,12,39^, climate change-induced extreme weather can potentially increase the prevalence of hyposalinity events in a broader distribution of corals and hamper coral recruitment processes. We showed that hyposalinity and decreased sperm concentration exerted substantial stress on the early life processes and stages of corals. These combined effects may lead to catastrophic failure in the first step of coral reproduction, which will further impair recovery of the already degraded coral populations. Active management strategies, for example restoration, should be prioritised to assist coral recovery in these inshore regions where depleted populations suffer low reproductive potential because of climate change-induced stressors.

## Methods

### Field collection for coral gametes and gamete preparation

The coral egg-sperm bundles were collected at A Ma Wan (AMW) in the Tung Ping Chau Marine Park (TPCMP, 22°32’ N, 114°25’ E) in the northeastern New Territories, Hong Kong. Scleractinian corals inside TPCMP are primarily distributed in shallow waters ranging from − 1 to − 4 m Chart Datum (CD)^64^. The gamete collection procedures followed that described in^36^. Egg-sperm bundles were collected from five tagged colonies of the two hard coral species, *Acropora tumida* and *Platygyra carnosa*, at approximately − 2 m CD depths in two consecutive spawning months in 2022. Egg-sperm bundles from each colony were collected separately with SCUBA using bundle collectors fitted with a 150-µm nylon mesh. After collection, the bundles were transferred to the Simon F. S. Li Marine Science Laboratory at the Chinese University of Hong Kong. The eggs and sperm were separated by gently stirring with plastic pipette tips. The sperm from the five coral colonies were pooled in a 500-mL glass beaker to create a sperm stock. The concentration in the sperm stock solution was determined using a haemocytometer with a compound light microscope. Subsequently, the eggs from each individual colony were transferred to separate plastic containers with a 125-µm plankton mesh bottom. The eggs were then washed with 100-µm filtered seawater for approximately 10 min to remove any residual sperm from each colony.

### Stress treatment experiments

The sperm stock solutions were serially diluted in 10-fold dilutions using 0.22-µm filtered seawater. The maximum concentrations of the sperm solutions for *A. tumida* and *P. carnosa* were 10^7^ and 10^6^ sperm mL^–1^, respectively, with the lowest concentration being 10^2^ sperm mL^–1^. Seawater solutions with salinities of 22, 26, 30, and 32 psu (ambient control level) were prepared by diluting natural seawater with milli-Q water. The salinity of the seawater used in each treatment was verified using a refractometer (Extech Instrument) before the start of the experiment. Different water baths were connected to heaters and chillers with a thermostat (Inkbird ITC-310T-B) to maintain the respective temperatures at 24, 27 (ambient control level), and 30 °C with a temperature fluctuation of ± 0.3 °C throughout the experiment. Underwater pumps were used to maintain circulation within each water bath, ensuring consistent temperature distribution. The temperatures of the water baths were monitored using the HOBO MX2202 Temperature/Light data logger (Fig. S2).

Fertilisation was initiated by adding approximately 150 eggs to each glass vial preloaded with each respective sperm concentration at different salinity combinations. These vials were incubated in water baths of different temperatures (Fig. S2). It must be noted that although extreme care was taken during sperm wash, some sperm might remain with the eggs, causing some background fertilisation. The background fertilisation could be caused by any unreported reproductive strategy, such as parthenogenesis. To ensure an accurate assessment of the true fertilisation success specifically attributable to the experimental treatments, two vials without any sperm (sperm-free control vials) were assigned to each treatment condition of different salinity and temperature, resulting in a total of 24 sperm-free control vials allocated for each species. The experiment was terminated once the embryos under the control condition reached the 8-cell stage, which typically occurred approximately 3 h after the mixing of gametes. At least 80 embryos were then haphazardly removed from each replicate vial and transferred into 1.5-mL Eppendorf tubes containing 2.5% glutaraldehyde buffered with 0.22-µm filtered seawater for fixing. Fifty fixed individuals from each tube were randomly sampled and assessed for fertilisation success, including the number of normal vs. abnormally developed eggs. Abnormally developing eggs were those that showed uneven, i.e. asymmetrical and irregular, cell cleavage patterns^12^.

### Statistical analysis

All statistical analyses were performed in R (v.4.3.1). Any background fertilisation observed in the sperm-free control vials corresponding to the different temperature and salinity treatments for each of the species was first subtracted from the fertilisation recorded for each treatment. For *A. tumida*, low background fertilisation was observed in the sperm-free control vials, with an average of 0.7 ± 1.3% (mean ± SD) fertilisation. For *P. carnosa*, the background fertilisation was higher, with an average of 10.3 ± 5.6% (mean ± SD) across different treatments.

Generalised Additive Mixed Models (GAMM) with a binomial error distribution were employed to assess the relationship between sperm concentration and fertilisation success at each treatment combination using the *mgcv* package^73^. The experimental vial was treated as an observational-level random effect^74^. Model diagnostics were performed using the *appraise* function from the *gratia* package^75^. Post-hoc pairwise comparisons were performed using package *emmeans* to test differences between temperatures within each salinity level, and between salinity levels within each temperature. Adjustment of *p*-values was made using the *p.adjust* function with the Holm method.

To further compare and analyse the non-linear patterns (curves) of fertilisation success at different sperm concentrations, two metrics were derived: maximum fertilisation success (F_max_) and the sperm concentration required to achieve 50% of the maximum fertilisation (EC_50_)^28^. This design also allows interpretation of whether eggs, sperm or the polyspermy block are being influenced by the stressors. The standard deviation of the metrics F_max_ and EC_50_ were estimated from each combination of temperature and salinity treatment using bootstrapping by resampling the residuals and refitting the GAMM 1000 times.

To investigate the combined effects of temperature and salinity on the proportion of abnormal embryo developed, Generalised Linear Mixed Models (GLMM) with a binomial error distribution were employed using the package *glmmTMB*^76^, with the inclusion of observational-level random effect of the experimental vials. As fertilisation success decreased with lower sperm concentrations (≤10^4^ and ≤10^3^ sperm mL^–1^ for *A. tumida* and *P. carnosa*, respectively), the resulting smaller sample sizes of fertilised eggs led to a large variation in the proportion of abnormal embryos. Thus, only the proportion of abnormal embryos from the higher sperm concentration treatments, i.e. sperm concentrations of 10^5^ and 10^6^ sperm mL^–1^, were considered for analysis. Likewise, under the lowest salinity level treatment (22 psu), the number of embryos was low due to the low fertilisation success for both species. Therefore, results from this treatment were not included in the analysis. Post-hoc pairwise comparisons were made for multiple comparisons to determine if there were significant differences in the proportion of abnormal embryos between the temperature treatments within each salinity level, and between salinity treatments within each temperature treatment level. Adjustment of *p*-values was again made using the *p.adjust* function with Holm method.

## Supplementary Information

Below is the link to the electronic supplementary material.

Supplementary Material 1

## Data Availability

The data from this study will be available based on reasonable requests through the corresponding author.
