# Supplementary material for: Effects of temperature, hyposalinity, and diminishing sperm concentration on fertilisation and embryonic development in Acropora tumida and Platygyra carnosa
Source: Sci Rep. 2026 Mar 20;16:14338. doi: 10.1038/s41598-026-41257-0 (PMC13144415; doi:10.1038/s41598-026-41257-0)
Supplement: Supplementary file 1 — Supplementary Material 1 [file 41598_2026_41257_MOESM1_ESM.docx]

## **Supplementary information**

**Table S1 Summary statistics of generalised additive mixed model (GAMM) examining the effect of temperature, salinity and sperm concentration on fertilisation success of *Acropora tumida* and *Platygyra carnosa*.** Bold *p*-values represent that statistical significance was found for the explanatory variables. edf: effective degrees of freedom.

| **Response variable** | **Species** | **Independent variables** | **df** | **Chi.sq** | ***p*-value** |
| --- | --- | --- | --- | --- | --- |
| Fertilisation success | *Acropora tumida* | Temperature | 2 | 35.97 | **<0.001** |
|  |  | Salinity | 3 | 632.87 | **<0.001** |
|  |  | Temperature x Salinity | 6 | 53.52 | **<0.001** |
|  |  | s(Sperm concentration, Sal) | 11.68 (edf) | 1026.94 | **<0.001** |
|  |  | s(Sperm concentration, Temp) | 2.27 (edf) | 19.92 | **<0.001** |
|  | *Platygyra carnosa* | Temperature | 2 | 81.84 | **<0.001** |
|  |  | Salinity | 3 | 266.99 | **<0.001** |
|  |  | Temperature x Salinity | 6 | 43.96 | **<0.001** |
|  |  | s(Sperm concentration, Sal) | 7.86 (edf) | 759.40 | **<0.001** |
|  |  | s(Sperm concentration, Temp) | 2.43 (edf) | 9.51 | **0.005** |

**Table S2** **Post-hoc pairwise comparisons of the GAMM between the predictor of salinity within each temperature treatment on the response of fertilisation success of *Acropora tumida* and *Platygyra carnosa*.** Bold *p*-values represent statistical differences in the pairwise comparisons.

| **Species** | **Contrast** | **Temp (°C)** | **Mean difference in fertilisation (proportions)** | **95% CI Lower** | **95% CI Upper** | **Adjusted *p*-values** |
| --- | --- | --- | --- | --- | --- | --- |
| *Acropora tumida* | Sal22 - Sal26 | 24 | -0.158 | -0.184 | -0.132 | **<.0001** |
|  | Sal22 - Sal30 |  | -0.447 | -0.486 | -0.407 | **<.0001** |
|  | Sal22 - Sal32 |  | -0.562 | -0.601 | -0.522 | **<.0001** |
|  | Sal26 - Sal30 |  | -0.289 | -0.330 | -0.248 | **<.0001** |
|  | Sal26 - Sal32 |  | -0.404 | -0.445 | -0.363 | **<.0001** |
|  | Sal30 - Sal32 |  | -0.115 | -0.163 | -0.068 | **<.0001** |
|  | Sal22 - Sal26 | 27 | -0.449 | -0.493 | -0.405 | **<.0001** |
|  | Sal22 - Sal30 |  | -0.775 | -0.805 | -0.745 | **<.0001** |
|  | Sal22 - Sal32 |  | -0.797 | -0.825 | -0.769 | **<.0001** |
|  | Sal26 - Sal30 |  | -0.326 | -0.374 | -0.277 | **<.0001** |
|  | Sal26 - Sal32 |  | -0.348 | -0.396 | -0.301 | **<.0001** |
|  | Sal30 - Sal32 |  | -0.023 | -0.057 | 0.011 | 0.5611 |
|  | Sal22 - Sal26 | 30 | -0.531 | -0.574 | -0.489 | **<.0001** |
|  | Sal22 - Sal30 |  | -0.694 | -0.730 | -0.659 | **<.0001** |
|  | Sal22 - Sal32 |  | -0.740 | -0.773 | -0.707 | **<.0001** |
|  | Sal26 - Sal30 |  | -0.163 | -0.213 | -0.113 | **<.0001** |
|  | Sal26 - Sal32 |  | -0.209 | -0.257 | -0.160 | **<.0001** |
|  | Sal30 - Sal32 |  | -0.046 | -0.088 | -0.003 | 0.3065 |
| *Platygyra carnosa* | Sal22 - Sal26 | 24 | -0.362 | -0.400 | -0.325 | **<.0001** |
|  | Sal22 - Sal30 |  | -0.423 | -0.461 | -0.384 | **<.0001** |
|  | Sal22 - Sal32 |  | -0.424 | -0.464 | -0.385 | **<.0001** |
|  | Sal26 - Sal30 |  | -0.060 | -0.109 | -0.012 | 0.4474 |
|  | Sal26 - Sal32 |  | -0.062 | -0.111 | -0.013 | 0.4474 |
|  | Sal30 - Sal32 |  | -0.002 | -0.051 | 0.048 | 1 |
|  | Sal22 - Sal26 | 27 | -0.315 | -0.354 | -0.276 | **<.0001** |
|  | Sal22 - Sal30 |  | -0.395 | -0.435 | -0.355 | **<.0001** |
|  | Sal22 - Sal32 |  | -0.378 | -0.418 | -0.337 | **<.0001** |
|  | Sal26 - Sal30 |  | -0.080 | -0.129 | -0.031 | 0.0716 |
|  | Sal26 - Sal32 |  | -0.062 | -0.112 | -0.013 | 0.4474 |
|  | Sal30 - Sal32 |  | 0.018 | -0.032 | 0.068 | 1 |
|  | Sal22 - Sal26 | 30 | -0.409 | -0.451 | -0.367 | **<.0001** |
|  | Sal22 - Sal30 |  | -0.462 | -0.503 | -0.420 | **<.0001** |
|  | Sal22 - Sal32 |  | -0.486 | -0.527 | -0.444 | **<.0001** |
|  | Sal26 - Sal30 |  | -0.052 | -0.103 | -0.002 | 0.7013 |
|  | Sal26 - Sal32 |  | -0.076 | -0.127 | -0.026 | 0.1307 |
|  | Sal30 - Sal32 |  | -0.024 | -0.074 | 0.025 | 1 |

**Table S3** **Post-hoc pairwise comparisons of the GAMM between the predictor of temperature within each salinity treatment on the response of fertilisation success of *Acropora tumida* and *Platygyra carnosa*.** Bold *p*-values represent statistical differences in the pairwise comparisons.

| **Species** | **Contrast** | **Salinity (psu)** | **Mean difference in fertilisation (proportions)** | **95% CI Lower** | **95% CI Upper** | **Adjusted *p*-values** |
| --- | --- | --- | --- | --- | --- | --- |
|  | Temp24 - Temp27 | 22 | -0.041 | -0.054 | -0.027 | **<.0001** |
| *Acropora tumida* | Temp24 - Temp30 |  | -0.024 | -0.036 | -0.013 | **0.0003** |
|  | Temp27 - Temp30 |  | 0.016 | 0.001 | 0.031 | 0.1279 |
|  | Temp24 - Temp27 | 26 | -0.332 | -0.380 | -0.284 | **<.0001** |
|  | Temp24 - Temp30 |  | -0.398 | -0.446 | -0.350 | **<.0001** |
|  | Temp27 - Temp30 |  | -0.066 | -0.124 | -0.009 | 0.1279 |
|  | Temp24 - Temp27 | 30 | -0.369 | -0.417 | -0.321 | **<.0001** |
|  | Temp24 - Temp30 |  | -0.272 | -0.324 | -0.221 | **<.0001** |
|  | Temp27 - Temp30 |  | 0.096 | 0.053 | 0.139 | 0.0002 |
|  | Temp24 - Temp27 | 32 | -0.276 | -0.322 | -0.231 | **<.0001** |
|  | Temp24 - Temp30 |  | -0.203 | -0.252 | -0.154 | **<.0001** |
|  | Temp27 - Temp30 |  | 0.073 | 0.035 | 0.112 | 0.002 |
| *Platygyra carnosa* | Temp24 - Temp27 | 22 | -0.052 | -0.068 | -0.036 | **<.0001** |
|  | Temp24 - Temp30 |  | -0.102 | -0.123 | -0.081 | **<.0001** |
|  | Temp27 - Temp30 |  | -0.050 | -0.074 | -0.027 | **0.0005** |
|  | Temp24 - Temp27 | 26 | -0.004 | -0.051 | 0.042 | 1 |
|  | Temp24 - Temp30 |  | -0.149 | -0.196 | -0.101 | **<.0001** |
|  | Temp27 - Temp30 |  | -0.144 | -0.192 | -0.097 | **<.0001** |
|  | Temp24 - Temp27 | 30 | -0.024 | -0.072 | 0.023 | 1 |
|  | Temp24 - Temp30 |  | -0.141 | -0.189 | -0.093 | **<.0001** |
|  | Temp27 - Temp30 |  | -0.117 | -0.164 | -0.069 | **<.0001** |
|  | Temp24 - Temp27 | 32 | -0.005 | -0.053 | 0.043 | 1 |
|  | Temp24 - Temp30 |  | -0.163 | -0.211 | -0.115 | **<.0001** |
|  | Temp27 - Temp30 |  | -0.159 | -0.207 | -0.110 | **<.0001** |

**Table S4 Summary statistics of generalised linear mixed model (GLMM) on the effect of temperature and salinity on the proportion of abnormal embryos of *Acropora tumida* and *Platygyra carnosa*.** Bold *p*-values represent that statistical significance was found for the explanatory variables.

| **Response variable** | **Species** | **Independent variables** | **df** | **Chi.sq** | ***p*-value** |
| --- | --- | --- | --- | --- | --- |
| Embryo abnormality | *Acropora tumida* | Temperature | 2 | 153.45 | **<0.001** |
|  |  | Salinity | 2 | 250.03 | **<0.001** |
|  |  | Temperature x Salinity | 4 | 63.64 | **<0.001** |
|  | *Platygyra carnosa* | Temperature | 2 | 354.75 | **<0.001** |
|  |  | Salinity | 2 | 308.25 | **<0.001** |
|  |  | Temperature x Salinity | 4 | 268.60 | **<0.001** |


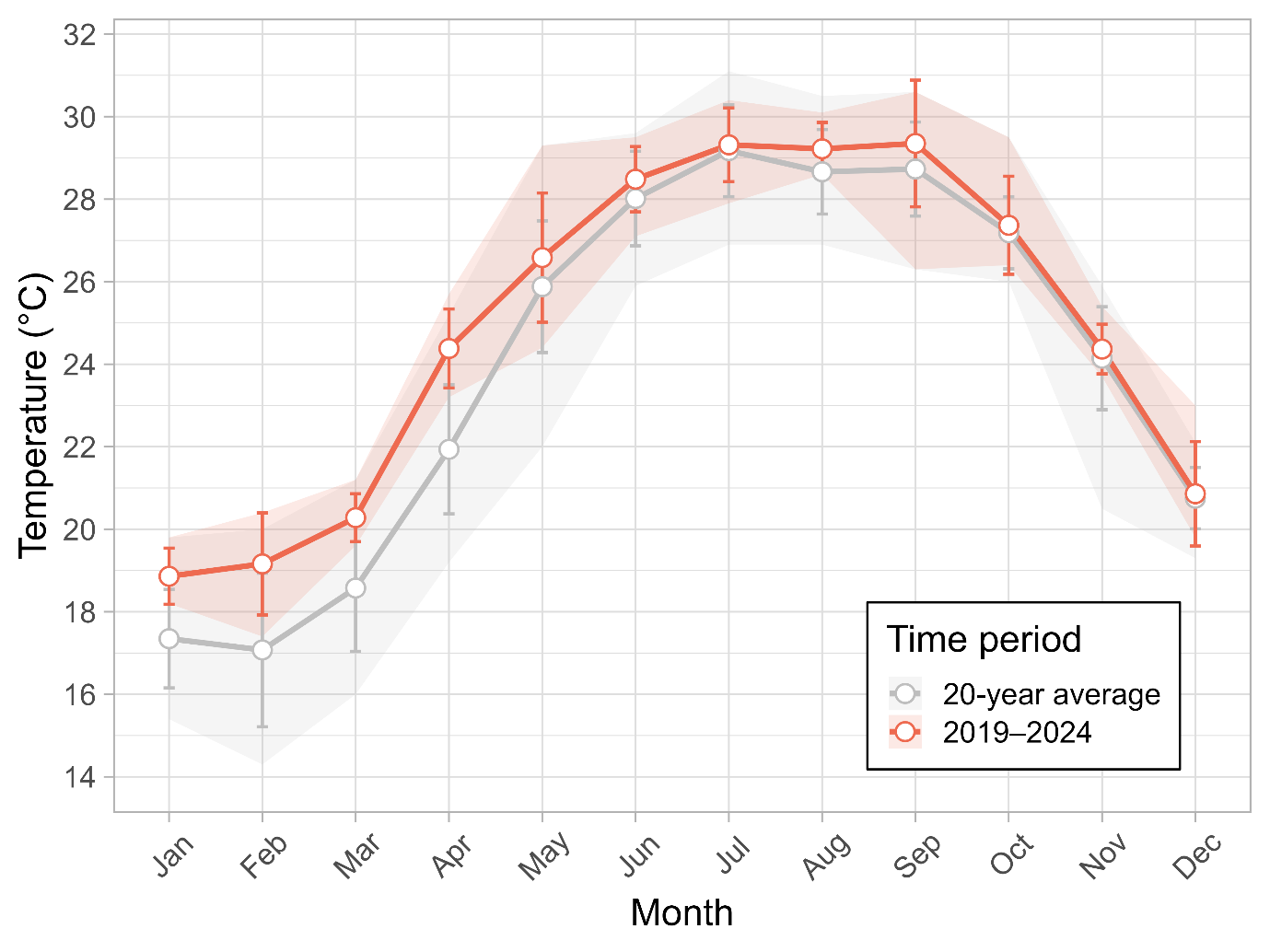


**Fig. S1 Comparison of historical sea surface temperature of Hong Kong waters in the recent five-year average (2019–2024) and the 20-year average (2002–2021).** Monthly sea surface temperature was measured in one of the water monitoring stations (MM5) in northeastern Hong Kong, which is the closest station to our coral sampling site (Tung Ping Chau Marine Park). Data were collected by the Environmental Protection Department of the HKSAR Government. The red solid line shows the mean sea surface temperature in the recent five years and salinity, and the grey solid line shows the 20-year mean sea surface temperature. The shaded areas show the maximum and minimum temperatures in each month.


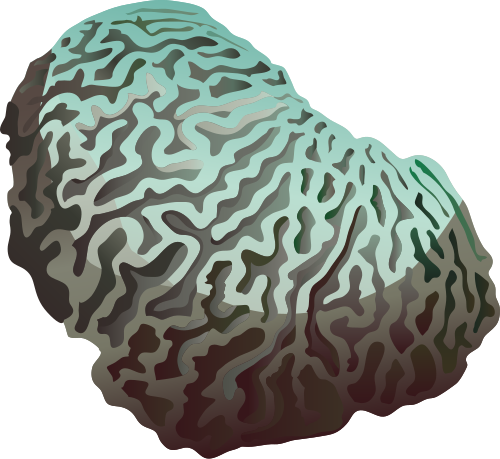

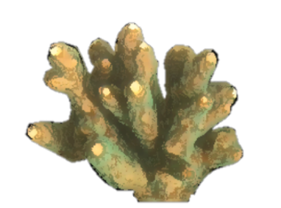

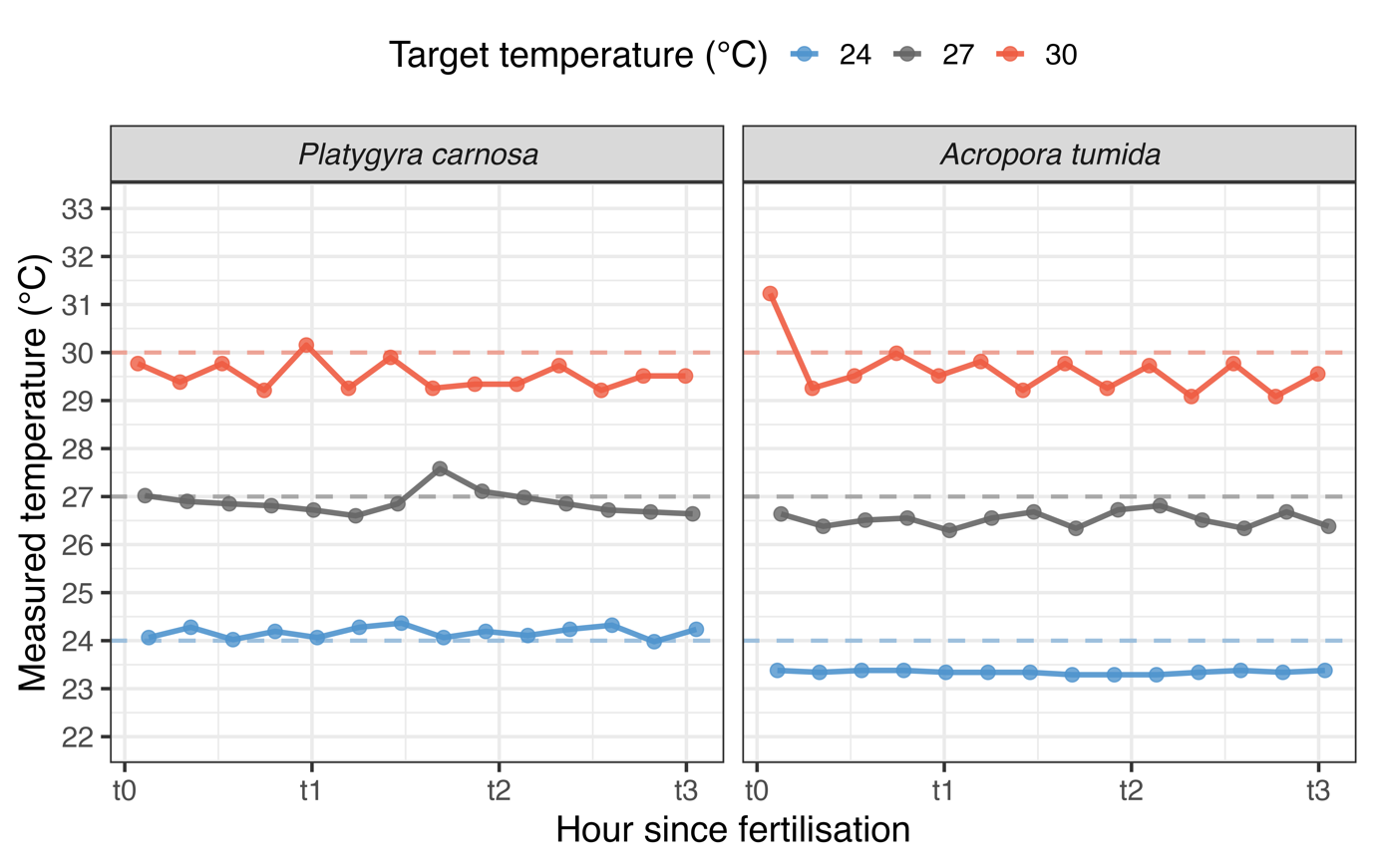


**Fig. S2 Real-time treatment temperature records from the start of the fertilisation and embryo development experiments.** The experiments were conducted in May and June on *Platygyra carnosa* and *Acropora tumida*. Arrows represent the start time of fertilisation. Temperature was monitored using HOBO MX2202 Temperature/Light Data Loggers, with a measurement interval set as 15 minutes.
